# Modulation of Toll-Like Receptor Signalling as a New Therapeutic Principle

**DOI:** 10.1155/2010/705612

**Published:** 2011-01-02

**Authors:** Philipp M. Lepper, Martha Triantafilou, Luke A. O'Neill, Natalija Novak, Hermann Wagner, Andrew E. Parker, Kathy Triantafilou

**Affiliations:** ^1^Department of Internal Medicine V—Pneumology, Allergology and Respiratory Intensive Care Medicine, Saarland University Hospital, 66421 Homburg, Germany; ^2^Infection and Immunity Group, Department of Child Health, School of Medicine, University of Cardiff, University Hospital of Wales, Heath Park, Cardiff CF14 4XN, UK; ^3^School of Biochemistry and Immunology, Trinity College Dublin, Dublin 2, Ireland; ^4^Department of Dermatology and Allergology, University of Bonn, 53012 Bonn, Germany; ^5^Institute for Medical Microbiology, Immunology and Hygiene, Technical University of Munich, 80333 Munich, Germany; ^6^Institute of Molecular Medicine, Trinity Centre for Health Sciences, St. James' Hospital, Dublin 8, Ireland

In the 20th century, immunologists focused much of their attention in deciphering the mechanisms of adaptive immunity. As a consequence, tremendous progress was made in the field of adaptive immunity, including tolerance, mechanisms of MHC restriction, the structure and function of MHC receptors, and development and activation of B- and T-cells. 

The reason why adaptive immunity was the major focus of immunologists is because adaptive immunity gives vertebrates immunity a memory and is most probably an important aspect of human evolution—responsible not only for enabling cognitive abilities but also general cultural accomplishments.

In contrast, innate immunity was sidelined. It was viewed as the most archaic of the two branches of immunity, simple and unsophisticated. Since the discovery of phagocytes by Ilya Mechnikov in 1882 and the investigations of Richard Pfeiffer, a fellow worker of Robert Koch, on “endotoxin,” the concept of innate immunity for almost 100 years was that of a static and nonspecific apparatus. It was seen as an undifferentiated system, engulfing and digesting invaders in contrast to the sophisticated framework of T- and B-cells that have elaborate clonal mechanisms to form a plethora of highly specific antibodies by DNA rearrangement. 

Almost twenty years ago, Charles Janeway changed our view of the innate immune system, by publishing “*Approaching the asymptote*” as part of the *Cold Spring Harbor Symposium* on immune recognition. In this publication, he predicted that there would be molecules that were encoded in the *germ line* which would recognize the presence of molecules produced by broad classes of pathogens. He called these molecules pattern recognition receptors (PRRs) and the ligands that they recognise, pathogen-associated molecular patterns (PAMPs).

Janeway's view was justified in the late 1990s with the discovery of Toll-like receptors (TLRs) and the verification that the innate immune system is actually highly specific, relying on germline-encoded pattern-recognition receptors (PRRs) that have evolved to detect components of foreign pathogens referred to as PAMPs. Over the years, research studies have shown that this system is highly specific in recognising microbial signatures; it has virtues that are equally specific and elaborate as the features of adaptive immunity; for example, TLR4 was found to recognise bacterial lipopolysaccharide (LPS) or endotoxin; TLR2 was found to recognise lipoteichoic acid (LTA) and peptidoglycan; TLR3 was able to sense double-stranded viral RNA; TLR5 was found to recognise bacterial flagellin; TLR7 and TLR8 to sense single stranded viral RNA, whereas TLR9 was found to delicately distinguish between methylated DNA from host DNA and unmethylated DNA from microorganisms. Subsequently, TLRs have been identified as operational centers for both innate and adaptive immunity. 

Cancer, infection, autoimmune, and allergic disorders involve various complex signaling pathways; however, data accumulates that TLRs are involved in all of these seemingly different entities. 

Their ability to initiate and propagate inflammation makes them attractive therapeutic targets. By understanding TLR-induced mechanisms, we can design more targeted therapeutic interventions for inflammatory disorders in the future. The ancient system of host defense of our cells is an outstanding target for intervention in the relentless efforts in finding cures. Pharmacological intervention in TLR pathways may potentially hold great therapeutic promise. Although for the past decades corticosteroids and in some instances antibodies were the mainstay of anti-inflammatory treatment, we are now at the stage of evaluating TLR pathway modifying molecules in human diseases; this targeted approach will bring more specificity to the treatment of a wide panel of disorders. 

Given the body of excellent literature on TLRs in disease, we can be very optimistic that targeting them will prove useful in human disease. In this special issue, we compiled 24 interesting papers on various aspects of human disease ranging from autoimmune disorders via dermatologic disease to cardiovascular and respiratory problems, demonstrating the impact of TLRs in a vast number of disorders and the as-yet unrealized therapeutic potential that is awaiting to be translated from the bench to the clinic.

